# Fear of cancer recurrence among caregivers of adolescents with malignant bone tumors in China: a cross-sectional survey

**DOI:** 10.3389/fpubh.2025.1608217

**Published:** 2025-09-24

**Authors:** Ye-jing Xuan, Qun Ye, Ying Ren, Yu-hong Yao, Zhan Wang, Xiu-qin Feng

**Affiliations:** ^1^Nursing Department, The Second Affiliated Hospital of Zhejiang University School of Medicine, Hangzhou, China; ^2^Department of Orthopedic Surgery, The Second Affiliated Hospital of Zhejiang University School of Medicine, Hangzhou, Zhejiang, China

**Keywords:** adolescents, Malignant bone tumor, FCR, caregivers, influencing factors

## Abstract

**Background:**

Fear of cancer recurrence (FCR) is not only present in cancer patients, but also in their caregivers, and caregivers' fear of cancer recurrence is greater than that of cancer patients. The purpose of this study was to explore the current status of cancer recurrence fear and the factors influencing it among caregivers of adolescents with malignant bone tumors, and to provide a theoretical basis for the development of effective interventions in the future.

**Methods:**

A total of 249 caregivers of adolescents with malignant bone tumors were selected for this study from two hospitals in Hangzhou, Zhejiang Province, from March 2023 to March 2025. The survey was conducted using the demographic questionnaire Fear of Progression Questionnaire – Short Form/Caregiver Version (FoP-Q-SF/C), the Simple Coping Style Questionnaire (SCSQ), and the Family Hardiness Index (FHI). Factors influencing FCR in caregivers of adolescents with malignant bone tumors were determined by univariate and logistic regression analyses.

**Results:**

The mean score for the FoP was 35.38 ± 7.66, and 62.2% (*n* = 155) of caregivers had high levels of fear of cancer recurrence. Logistic regression analyses showed that female sex [odds ratio (OR) = 2.690, 95% confidence interval (CI): 1.187–6.098, *P* = 0.018], living in a rural area (OR = 2.450, 95% CI: 1.199–5.009, *P* = 0.014), burden of care (OR = 2.832, 95% CI: 1.649–4.863, *P* < 0.001), economic burden (OR = 2.207, 95% CI: 1.079–3.810, *P* = 0.004), family hardiness index (OR = 0.857, 95% CI: 0.787–0.932, *P* < 0.001) and positive coping style (OR = 0.887, 95% CI: 0.806–0.976, P = 0.014) had independent influences on FCR.

**Conclusions:**

FCR is a huge burden for caregivers of adolescents with malignant bone tumors. Healthcare professionals should adopt targeted disease-related health education for caregivers, help caregivers enhance the family hardiness index, guide caregivers to adopt positive coping styles, and increase caregivers' confidence in facing cancer, thereby reducing the FCR.

## 1 Introduction

The Global Cancer Report 2020 (1) shows that there were 19.29 million cases of cancer, with nearly 10 million deaths, and 28.4 million new cases of cancer are expected in 2040. According to the statistics of the Union for International Cancer Control (UICC), there are about 105,000 new cases of adolescent cancer every year, its incidence is growing at a rate of 1.5% per year, and the burden of the disease shows an increasing trend year over year (2).

Although the majority of cancers that cause death are lung, prostate, and breast cancers, primary malignant tumors of the bones and joints have been ranked as the third leading underlying cause of death for cancer patients under 20 years of age (3). Osteosarcoma is the most common primary malignant bone tumor in adolescents, with a 5-year survival rate of 60–70% in patients without metastases but only 20% in those with metastases, and recurrence in up to 35% of patients (4, 5).

Given the high recurrence rate after surgery, the fear of cancer recurrence (FCR) has become a major psychological problem plaguing adolescent patients with malignant bone tumors and their caregivers (6). Simard et al. (7) found that 39–97% of cancer patients reported having FCR (average 73%). The study showed that fear of cancer recurrence is not only prevalent among patients, but also among their caregivers, and that caregivers have higher levels of FCR than patients (8). In China, the main task of nursing adolescent patients with malignant bone tumors often falls on the caregiver. In the face of the high cost of surgical treatment and the side effects (nausea, vomiting, fatigue) of long-term chemotherapy and radiotherapy, the caregiver not only faces a huge economic burden, but also a heavy nursing burden, which may lead to anxiety and FCR (9). Dwelling on the FCR for a long time will not only increase the psychological burden on the caregiver, but also may adversely affect their physical health, psychological state and quality of life, thus affecting the recovery of the patients in their care (10).

Currently, most studies on caregiver FCR have focused on ovarian (11), brain (8), hematologic (12), and breast (13) cancer, but the research on caregivers' FCR is often ignored. Therefore, to investigate the factors influencing FCR in the caregivers of adolescents with malignant bone tumors will provide a theoretical scientific basis for formulating interventions to reduce FCR in the future.

## 2 Materials and methods

### 2.1 Study design and participants

Between March 2023 and March 2025, 249 caregivers of adolescents with malignant bone tumors were recruited from the bone oncology departments of two general hospitals in Hangzhou, Zhejiang Province, China. This study adopted a convenience sampling method and caregivers were considered qualified if they met the following requirements.

Inclusion criteria: (1) Caregivers of adolescent patients with a definitive diagnosis of malignant bone tumors, including parents, relatives or friends; (2) Daily care duration ≥ 4 h and total duration of care exceeding 3 months; (3) The caregiver understood the patient's condition and family situation; and (4) The caregiver volunteered and was able to complete the test independently.

Exclusion criteria: (1) Caregivers who were paid; (2) Caregivers with serious heart, brain, lung, liver, kidney and other diseases; and (3) Caregivers with severe hearing and speech disorders.

According to the requirements of statistical variable analysis, the sample size should be 5 to 10 times the number of variables (14). In this study, 18 variables were statistically analyzed and the incidence of FCR among caregivers of adolescents with malignant bone tumors was 62%. Assuming 10% invalid questionnaires, the minimum sample size for this study was: 18 × 5 × (1 + 10%) ÷ 62% = 160, and the maximum sample size was: 18 × 10 × (1 + 10%) ÷ 62% = 319. The researchers sent 260 questionnaires to caregivers of adolescents with malignant bone tumors, and, after excluding 11 invalid questionnaires, a total of 249 valid questionnaires were recovered, with an effective recovery rate of 95.8%.

### 2.2 Procedures

Prior to the investigation, informed consent was obtained from the ethics committee of the hospital, the director of the bone tumor department and the head nurse. After explaining the purpose, significance and methods of this study to the caregivers of adolescents with malignant bone tumors who met the inclusion criteria, the investigators informed them that the survey followed the principles of fairness and confidentiality, and issued questionnaires after the caregivers had signed the informed consent form. The questionnaire was filled in by the caregivers according to their own situation.

### 2.3 Measures

#### 2.3.1 Sociodemographic characteristics

The questionnaire on sociodemographic characteristics was designed by the researchers. Data included the caregiver's age, sex, place of residence, education level, relationship with the patient, religious belief, working status, number of children, the burden of care, economic burden, degree of understanding of the disease, and own health status.

#### 2.3.2 FoP-Q-SF/C

The Fear of Progression Questionnaire – Short Form/Caregiver Version was compiled by Mehnert et al. (15) in 2006, revised by Peng et al. (16) in 2021, and is used to measure FCR levels in caregivers. It includes 12 items, and the total score ranges from 12 to 60, using a 5-point Likert scale of 1 (“not at all”), 2 (“rarely”), 3 (“sometimes”), 4 (“often”), and 5 (“always”). A score ≥ 34 indicates a high degree of FCR in caregivers. Cronbach's α coefficient for the scale was 0.92.

#### 2.3.3 FHI

The Family Hardiness Index was compiled by Mccubbin et al. (17) in 1996, revised by Liu et al. (18) in 2014, and is used to measure family resilience. It includes 20 items, and the total score ranges from 20 to 80, using a 4-point Likert scale of 1 (“strongly disagree”), 2 (“disagree”), 3 (“agree”), and 4 (“strongly disagree”). Cronbach's α coefficient for the scale was 0.80.

#### 2.3.4 SCSQ

The Simple Coping Style Questionnaire was compiled by Folkman et al. (19) in 1988 and revised by Xie et al. (20) in 1998. There are two subscales, the positive coping style scale and negative coping style scale; the positive coping style scale was adopted in this study. The positive coping style scale includes 12 items, and the total score ranges from 0 to 36, using a 4-point Likert scale of 0 (“never”), 1 (“seldom”), 2 (“often”), and 3 (“always”). The higher the score, the more inclined the caregiver is to adopt a positive coping style. Cronbach's α coefficient for the scale was 0.89.

### 2.4 Statistical analysis

Data were analyzed using SPSS 26.0 (Armonk, NY: IBM Corp). Descriptive statistics were used to analyze demographic and clinical characteristics, categorical variables were expressed as frequency and percentage, and continuous variables were expressed as mean ± standard deviation. The *t* test and χ^2^ test were used for univariate analysis, and the results with statistical significance in the univariate analysis (*P* < 0.05) were analyzed by multivariate logistic regression.

### 2.5 Ethical consideration

This study was approved by the Ethics Committee of the Second Affiliated Hospital of Zhejiang University School of Medicine (Approval No. I2023053). This study follows the principles of voluntary participation, non-harm and confidentiality. Before the investigation, the researcher explained the purpose and significance of this study to the caregivers, and the caregivers signed the relevant informed consent form.

## 3 Results

### 3.1 Caregivers information and characteristics

We recruited 249 caregivers to this study. Among the respondents, 65.1% were 40–59 years old (*n* = 162), and 75.9% were female (*n* = 189); 51.4% of caregivers were from urban areas and 48.6% from rural areas. The majority of caregivers (69.1%) were mothers and only 11% were other caregivers (including relatives and friends). Most caregivers had a middle school education or below. In addition, 19.7% (*n* = 49) of caregivers had no knowledge of the disease, 59.0% (*n* = 147) had partial knowledge of the disease, and 21.3% (*n* = 53) had complete knowledge of the disease (Table 1).

**Table 1:** Univariate analysis of factors influencing FCR in caregivers.

**Variables**	**Total (*n =* 249) N (%) M (D)**	**Low FCR group (*n =* 94) N (%) M (D)**	**High FCR group (*n =* 155) N (%) M (D)**	***P***
**Age**				
20~39 years	70 (28.1)	28 (29.8)	42 (27.1)	0.131
40~59 years	162 (65.1)	56 (59.6)	106 (68.4)	
≥ 60 years	17 (6.8)	10 (10.6)	7 (4.5)	
**Gender**				
Male	60 (24.1)	36 (38.3)	24 (15.5)	< 0.001
Female	189 (75.9)	58 (61.7)	131 (84.5)	
**Place of residence**				
Urban	128 (51.4)	63 (67.0)	65 (41.9)	< 0.001
Rural	121 (48.6)	31 (33.0)	90 (58.1)	
**Education level**				
Elementary School	40 (16.1)	15 (16.0)	25 (23.0)	0.389
Middle School	110 (44.2)	37 (39.4)	73 (50.0)	
High School	74 (29.7)	29 (30.9)	45 (23.0)	
University and above	25 (10.0)	13 (13.8)	12 (4.0)	
**Relationship with patients**				
Father	45 (18.1)	21 (22.3)	24 (15.5)	0.146
Mother	172 (69.1)	58 (61.7)	114 (73.5)	
Other caregivers	32 (11.0)	15 (16.0)	17 (11.0)	
**Religious belief**				
No	191 (76.7)	68 (72.3)	123 (79.4)	0.204
Yes	58 (23.3)	26 (27.7)	32 (20.6)	
**Working state**				
On-the-job	114 (45.8)	49 (52.1)	65 (41.9)	0.118
Nonworking	135 (54.2)	45 (47.9)	90 (58.1)	
**Number of children**				
≤ 1	135 (54.2)	48 (51.1)	87 (56.11)	0.303
2	108 (43.4)	42 (44.7)	66 (42.6)	
≥3	6 (2.4)	4 (4.3)	2 (1.3)	
**The burden of care**				
Light	62 (24.9)	45 (47.9)	17 (11.0)	< 0.001
Medium	107 (43.0)	37 (39.4)	70 (45.2)	
Heavy	80 (32.1)	12 (12.8)	68 (43.9)	
**The burden of economy**				
Light	60 (24.1)	46 (48.9)	14 (9.0)	< 0.001
Medium	113 (45.4)	38 (40.4)	75 (48.4)	
Heavy	76 (30.5)	10 (10.6)	66 (42.6)	
**Degree of disease understanding**				
Ignorant	49 (19.7)	17 (18.1)	32 (20.6)	0.160
Partial	147 (59.0)	51 (54.3)	96 (61.9)	
Full	53 (21.3)	26 (27.6)	27 (17.5)	
**Self-health status**				
Poor	21 (8.4)	4 (4.3)	17 (11.0)	0.178
General	130 (52.2)	52 (55.3)	78 (50.3)	
Good	98 (39.4)	38 (40.4)	60 ( 38.7)	
**Family Hardiness Index**	53.85 ± 5.50	57.31 ± 4.42	51.75 ± 5.02	< 0.001
**Positive coping style**	16.86 ± 4.56	19.45 ± 4.33	15.29 ± 3.94	< 0.001

### 3.2 FHI, and SCSQ scores in caregivers

Among the caregivers, 62.2% (*n* = 155) experienced a high level of FCR and 37.8% (*n* = 94) experienced a low level of FCR (FoP-Q-SF ≥ 34). The mean FHI score of the caregivers was 53.85 ± 5.50, with 57.31 ± 4.42 in the low FCR group and 51.75 ± 5.02 in the high FCR group. The mean positive coping score of the caregivers was 16.86 ± 4.56; the mean score of the low FCR group was 19.45 ± 4.33, and the mean score of the high FCR group was 15.29 ± 3.94 (Table 1).

### 3.3 Univariate analysis of factors influencing FCR in caregivers

The results of the univariate analysis showed that sex, place of residence, the burden of care, the economic burden, family hardiness index and positive coping style were the main factors influencing caregiver FCR (*P* < 0.05). In particular, the FCR level of female caregivers was higher than that of male caregivers (*P* < 0.001), the FCR level of those living in rural areas was higher than that of those living in urban areas (*P* < 0.001), and the FCR level of those with a heavy caregiver burden was higher than that of those with a light caregiver burden (*P* < 0.001). The level of FCR in caregivers who experienced a heavy financial burden was higher than that of caregivers with a low financial burden (*P* < 0.001) (Table 1).

### 3.4 Multifactorial analysis of factors influencing FCR in caregivers

Table 2 shows the scale independent variable assignment. Logistic regression analysis revealed that female sex [odds ratio (OR) = 2.690, 95% confidence interval (CI): 1.187–6.098, *P* = 0.018], living in a rural area (OR = 2.450, 95%CI: 1.199–5.009, *P* = 0.014), the burden of care (OR = 2.832, 95%CI: 1.649–4.863, *P* < 0.001), and the economic burden (OR = 2.207, 95%CI: 1.279–3.810, *P* = 0.004) were all associated with a higher level of FCR. A higher family hardiness index (OR =0.857, 95%CI: 0.787–0.932, *P* < 0.001) and positive coping style (OR = 0.887, 95%CI: 0.806–0.976, *P* < 0.001) were associated with a lower level of FCR (Table 3).

**Table 2:** Assignment of independent variables.

**Variant**	**Assignment method**
Age	20 ~ 39 years = 1, 40 ~ 59 years = 2, ≥60 years = 3
Gender/sex	Male = 1, Female = 2
Place of residence	Urban = 1, Rural = 2
Education level	Elementary School = 1, Middle School = 2, High School = 3, University and above = 4
Relationship with patients	Father = 1, Mother = 2, Other caregivers = 3
Religious belief	No = 1, Yes = 2
Working state	On the job = 1, nonworking = 2
Number of children	1 = 1, 2 = 2, ≥3 = 3
The burden of care	Light = 1, Medium = 2, Heavy = 3
The burden of economy	Light = 1, Medium = 2, Heavy = 3
Degree of disease understanding	Ignorant = 1, partial = 2, full = 3
Self-health status	Poor = 1, general = 2, good =3
Family Hardiness Index	Original value input
Positive coping style	Original value input

**Table 3:** Results of Logistic regression analysis of factors influencing FCR.

**Variables**	**β**	**SE**	**Wald**	***P***	***OR***	**Multivariable 95% CI for OR**	
						**Lower**	**Upper**
**Gender (Ref: Male)**					1.000		
Female	0.989	0.418	5.615	0.018	2.690	1.187	6.098
**Place of residence (Ref: Rural)**					1.000		
Rural	0.896	0.365	6.033	0.014	2.450	1.199	5.009
**The burden of care**	1.041	0.276	14.234	< 0.001	2.832	1.649	4.863
**The burden of economy**	0.792	0.278	8.085	0.004	2.207	1.279	3.810
**Family Hardiness Index**	−0.155	0.043	12.857	< 0.001	0.857	0.787	0.932
**Positive coping style**	−0.120	0.049	6.091	0.014	0.887	0.806	0.976

## 4 Discussion

### 4.1 A high incidence of FCR in caregivers of adolescents with malignant bone tumors

This study investigated the main factors influencing FCR in caregivers of adolescents with malignant bone tumors and found that FCR is prevalent in caregivers. Among the 249 caregivers surveyed, the mean caregiver FCR score was 35.38 ± 7.66 points, and 62.2% of caregivers scored ≥34 points. Li et al. (21) conducted a survey of 234 caregivers of patients with colorectal cancer, showing that 61.5% of caregivers experienced high levels of FCR, similar to the findings in this study. It can be seen that FCR not only exists in adolescent patients with malignant bone tumors, but also is a common psychological problem faced by many caregivers. Caregivers not only have to bear the pain of the disease itself, but also take care of the patient and the whole family, and even face the blow of losing loved ones, all factors that induce the psychological fear of cancer recurrence. Therefore, in their usual work, medical staff should not only pay attention to the psychological state of the patient, but also enquire about the negative emotions of the caregiver. They should provide targeted psychological guidance to the caregiver, so that the caregiver can actively face the pain caused by the disease, in order to reduce the FCR in caregivers of adolescents with malignant bone tumors.

### 4.2 Factors influencing FCR levels in caregivers

#### 4.2.1 Sex

The results of this study indicate that sex is a major factor influencing FCR in caregivers of adolescents with malignant bone tumors: female caregivers had a higher level of FCR than male caregivers, which is consistent with the findings of Maguire et al. (22). The reason may be related to the high sensitivity and empathy of female caregivers. In the face of cancer, women have a heavier psychological burden and are more likely to develop negative emotions such as anxiety and depression, which leads to an increase of FCR. In addition, when female caregivers face cancer, they may be more inclined to express their FCR and seek outside assistance. Men may feel ashamed to express their fears or concerns to others when faced with cancer (23). However, the results of Muldbucker et al. (24), who studied FCR in caregivers of breast cancer patients were different from this study. In Muldbucker's study, male caregivers had a higher level of FCR, and on analyzing the possible reasons it was found that male caregivers were often not skilled enough in taking care of patients, and their caring roles were likely to be lacking and they were prone to more pain. Therefore, the effect of sex on caregiver FCR in different studies is controversial, and more research is needed to explore the effect of sex on caregiver FCR.

#### 4.2.2 Place of residence

Our study found that caregivers living in rural areas had a higher level of FCR, and Maguire et al. (22) found the same. The reason may be that caregivers living in rural areas tend to have low income and poor disease cognition, resulting in uncertainty about the disease and increasing FCR, whereas caregivers living in urban areas have excellent public medical resources and can seek better care (25). However, Zhao et al. (26) found no relationship between residence and FCR. Therefore, more multicenter studies are needed to explore the relationship between residence and FCR among caregivers of adolescents with malignant bone tumors.

#### 4.2.3 The burden of care

We found that caregiving burden affects the FCR of caregivers, and the degree of FCR of caregivers with heavy caregiving burden is higher than that of caregivers with light caregiving burden, similar to the results of Padova et al. (27). The reason may be that the condition of adolescent patients with malignant bone tumors is complicated, the treatment process is cumbersome, and the caregivers bear huge physical and psychological pressure during the care process (28). Excessive pressure further increases the anxiety and pain of the caregivers, so that they are more afraid of the recurrence of cancer. Therefore, medical workers should assess the burden of care borne by caregivers, provide them with targeted support, and help them establish an effective family support system through psychological counseling and peer support.

#### 4.2.4 The economic burden

We found that the financial burden was the main factor influencing FCR in the caregivers of adolescents with malignant bone tumors: a heavy financial burden will affect the caregivers' FCR, similar to the results of a study by Li et al. (13). The reason may be that the disease caused by a malignant bone tumor in an adolescent lasts a long time and is prone to relapse, and the treatment process costs a lot of money (29). For families with relatively comfortable economic conditions, the economic burden they face is relatively light and they can afford it, but for families with difficult economic conditions, they need to bear a huge economic burden. This will further increase their mental pressure, and they will be in a state of extreme tension and anxiety for a long time, fearing the recurrence of the cancer. Therefore, healthcare workers should focus on families with difficult financial conditions, choose the right treatment plan for patients according to their economic status, and help them get more financial support from charities to reduce their financial burden and reduce FCR.

#### 4.2.5 Family hardiness

We found that family hardiness was negatively associated with FCR (β = −0.155, *P* < 0.001), similar to the results of the study by Hu et al. (30). The Family Hardiness Index is an important tool for assessing the entire family's ability to resist pressure and adapt. Family hardiness, also known as family resilience (including tenacity, strength, and optimism), is an important aspect of positive psychology (31). It can help caregivers face cancer in a positive state; the more resilient a caregiver is, the lower their FCR. However, in contrast to the results of Ye et al. (32), our study showed that the mean FHI score of caregivers was 53.85 ± 5.50; Ye et al. reported that the mean FHI of caregivers was 59.21 ± 8.57, which may be related to the 10-year survival rate of thyroid cancer patients in comparison with that of adolescent patients with malignant bone tumors.

#### 4.2.6 Positive coping style

This study shows that positive coping styles have a significant negative impact on caregivers' FCR, confirming previous findings that adopting positive coping styles helps reduce FCR (33). There is no denying that positive coping styles are crucial for caregivers. Positive coping styles can help caregivers deal with stress responses triggered by disease, help caregivers cope with various difficulties with a positive attitude, and establish effective communication to help them reintegrate into society, build confidence in fighting cancer, and reduce FCR. Kang et al. (34) proposed that aerobic running on a treadmill three times a week for 12 weeks can effectively relieve patients' anxiety about their disease, improve the level of positive coping, and reduce FCR. Therefore, it is incumbent on healthcare workers to provide caregivers with clear caregiving advice that guides them to adopt more positive coping styles and reduce FCR.

### 4.3 Clinical implications

This study suggests that FCR is a common psychological problem among caregivers of adolescents with malignant bone tumors. This study identified that a number of factors (sex, place of residence, the burden of care, the economic burden, family hardiness, positive coping style) were associated with FCR in caregivers of malignant bone tumors. Clinicians should pay more attention to caregivers who are female, live in rural areas, have heavy financial burden, heavy caring burden, low family resilience, and low positive coping style. To reduce the FCR of caregivers, targeted interventions should be formulated according to different characteristics of caregivers in the future.

### 4.4 Study limitations

There are some limitations to this study. This study was a cross-sectional survey of caregivers of adolescents with malignant bone tumors, and there was a lack of follow-up for different time periods. It is suggested that longitudinal studies should be conducted to investigate the trajectory of FCR in caregivers of adolescents with malignant bone tumors. In addition, this study was a quantitative study, and qualitative research should be carried out in the future.

## 5 Conclusions

In this study, the incidence of high FCR among caregivers of adolescents with malignant bone tumors was 62.2%. The high FCR in caregivers of adolescents with malignant bone tumors who are female, live in rural areas, have a heavy care burden, heavy economic burden, low family resilience, and lack of a positive coping style suggests that medical staff should pay attention to such caregivers, evaluate the psychological status of this group, and carry out preventive interventions to reduce the occurrence of FCR.

## Data Availability

The original contributions presented in the study are included in the article/supplementary material, further inquiries can be directed to the corresponding author.
